# Perceiving pitch absolutely: Comparing absolute and relative pitch possessors in a pitch memory task

**DOI:** 10.1186/1471-2202-10-106

**Published:** 2009-08-27

**Authors:** Katrin Schulze, Nadine Gaab, Gottfried Schlaug

**Affiliations:** 1 Department of Neurology, Music and Neuroimaging Laboratory, Beth Israel Deaconess Medical Center and Harvard Medical School, 330 Brookline Avenue, Boston, MA, 02215, USA

## Abstract

**Background:**

The perceptual-cognitive mechanisms and neural correlates of Absolute Pitch (AP) are not fully understood. The aim of this fMRI study was to examine the neural network underlying AP using a pitch memory experiment and contrasting two groups of musicians with each other, those that have AP and those that do not.

**Results:**

We found a common activation pattern for both groups that included the superior temporal gyrus (STG) extending into the adjacent superior temporal sulcus (STS), the inferior parietal lobule (IPL) extending into the adjacent intraparietal sulcus (IPS), the posterior part of the inferior frontal gyrus (IFG), the pre-supplementary motor area (pre-SMA), and superior lateral cerebellar regions. Significant between-group differences were seen in the left STS during the early encoding phase of the pitch memory task (more activation in AP musicians) and in the right superior parietal lobule (SPL)/intraparietal sulcus (IPS) during the early perceptual phase (ITP 0–3) and later working memory/multimodal encoding phase of the pitch memory task (more activation in non-AP musicians). Non-significant between-group trends were seen in the posterior IFG (more in AP musicians) and the IPL (more anterior activations in the non-AP group and more posterior activations in the AP group).

**Conclusion:**

Since the increased activation of the left STS in AP musicians was observed during the early perceptual encoding phase and since the STS has been shown to be involved in categorization tasks, its activation might suggest that AP musicians involve categorization regions in tonal tasks. The increased activation of the right SPL/IPS in non-AP musicians indicates either an increased use of regions that are part of a tonal working memory (WM) network, or the use of a multimodal encoding strategy such as the utilization of a visual-spatial mapping scheme (i.e., imagining notes on a staff or using a spatial coding for their relative pitch height) for pitch information.

## Background

Absolute Pitch (AP) is defined as the ability to identify any pitch of the Western musical scale without an external reference tone [1,2]. Although AP has fascinated researchers over several decades, the underlying perceptual/cognitive mechanisms as well as the neural correlates of this unique ability remain unclear [3-9]. Several studies [10-12] suggested that AP musicians perceive tones more categorically than subjects without AP, who perceive tones on a category independent, physical continuum.

On a structural brain level, the planum temporale (PT) has been suggested as a possible anatomical marker for AP, since an increased leftward PT asymmetry was associated with the AP phenotype [3,4,13,14]. There has been some support from functional studies for a particular involvement of the PT when AP musicians were asked to process pitch information. Ohnishi et al. [15] suggested distinct brain activities within the posterior superior temporal region when AP musicians were compared with non-AP musicians in a passive music listening task. Other functional imaging studies found differences predominantly in extratemporal brain regions. Zatorre and colleagues [4] compared musicians with and without AP in a tone perception task and observed increased activation in a left posterior dorsolateral frontal region in AP musicians. The non-AP musicians in this study revealed a similar activation in this location during an interval classification task, suggesting that this region could be involved in verbal labeling or other associations. The authors also found this region to be activated when non-musicians were taught to associate numbers with certain chords [16] even further suggesting that this region might be involved in associative learning.

In the search for a neural substrate of AP, brain regions involved in short-term or working memory have also received attention. This was triggered by electrophysiological experiments showing an absent or smaller P300 component in AP musicians (compared to non-AP musicians) in a pitch memory task [17-20]. The P300 is an evoked response thought to reflect working memory (WM) processes [17]. The smaller or absent P300, although still debated [21,22], has been interpreted as an indication that AP musicians may not, or to a lesser degree, require a WM update during a pitch memory task. Two models of short-term memory (STM) and working memory (WM) might be of relevance here. The Baddeley and Hitch [23] WM model describes a phonological loop, which can be further subdivided into a passive storage component (phonological store) and an active rehearsal component (articulatory rehearsal process) [24]. The phonological rehearsal process might involve the posterior inferior frontal gyrus (Broca's area) and associated premotor areas [25-28], while the phonological store seems to rely on superior and in particular the inferior parietal lobule [25,27,29]. Although the Baddeley and Hitch [23,24] WM model does not specifically exclude the processing of non-verbal information from their WM model, the phonological loop was implemented into this model to explain WM processes related to verbal information. On the other hand, Deutsch has put forward a model of pitch memory [30], suggesting that a separate system for non-verbal, tonal pitch information must exist, since the memory for tonal pitch can be disturbed by other tones, but only minimally by verbal information [31]. There are however studies suggesting that verbal and tonal stimuli are processed in the same WM system [32,33].

There are very few imaging studies that have compared WM for verbal and nonverbal information. A recent study by Koelsch et al. [34] suggested that rehearsal of verbal and tonal material leads to similar and overlapping activations in regions of the brain that have been traditionally associated with verbal WM only. Furthermore, studies that have only used tonal WM tasks have found activations in regions that are traditionally associated with verbal WM [35-37]. Thus, it is possible that the tonal memory system shares some resources with the verbal memory system.

Several neurophysiological studies found differences between AP and non-AP musicians during the perceptual phase of tonal information suggesting that the perception and early auditory encoding might be different if one possesses AP. Using magnetoencephalography (MEG), Hirata et al. [38] found spatial differences in the N100m dipole moments during tone perception tasks between participants who did posses AP and those who did not. Itoh et al. [39] showed in an EEG study a unique left posterior temporal negativity in AP possessors ("AP negativity") with a latency of 150 ms in both listening and pitch-naming tasks suggesting that this unique signal is triggered by pitch input in AP possessors irrespective of the task performed. Wu et al. [40] observed greater activity in AP than in non-AP musicians in left and right auditory regions during a pitch labeling task. We suggest that the results of these experiments highlight two components of AP. One component is the perceptual and early encoding which might differ between AP and non-AP. AP possessors might perceive and encode tonal information as belonging to pre-defined pitch chroma categories [10,11]. The second component is the labeling of these pitch chroma categories, which might be the result of a learned association. There are other examples in the auditory domain in which information is perceived as belonging to pre-defined categories. An example for such a process is work by Belin et al. [41] who reported stronger activation in the STS when participants listened to vocal sounds compared to environmental sounds assuming that participants had some pre-existing codes for vocal sounds.

Our aim was to contrast AP with non-AP musicians using an established pitch memory task [37,42,43] in order to reveal possible differences in the neural correlates of early encoding and short-term storage of tonal information [44]. A sparse temporal fMRI method with clustered volume acquisition [37,45] allowed us to separate the early perceptual (encoding) phase from the later short-term memory phase of this pitch memory task. We hypothesized that the difference between AP and non-AP musicians would be reflected in a stronger activation in AP musicians of the left PT and/or left STG/STS complex as candidate brain regions for receiving and encoding tonal information in categories [41], and possibly in inferior frontal regions if a verbal code for the pitch categories was rehearsed in order to perform the pitch memory task [24]. Furthermore, we hypothesized that non-AP musicians might rely stronger on brain regions belonging to a tonal memory system which might involve regions in the temporal and parietal lobes [37].

## Methods

### Participants

10 AP musicians and 10 non-AP musicians (age range: 18–40) provided written informed consent and participated in this study that was approved by the institutional review board of the Beth Israel Deaconess Medical Center. None of our participants reported any neurologic or psychiatric disorder. AP and non-AP musicians were matched for handedness using a standard handedness questionnaire [46] and an index finger tapping test to assess hand dexterity [47]. This 12-task inventory [46] defines handedness according to the hand preferred for each task. Consistent right-handers (CRH) or consistent left-handers (CLH) are those that perform all of the six primary tasks (writing, throwing, using a tennis racket, striking a match, using a hammer, and using a toothbrush) with either the right or left hand. According to this questionnaire, 18 participants were consistent right-handers (9 AP and 9 non-AP), and 2 participants were mixed-handers (1 AP and 1 non-AP). We decided to enroll only male musicians in this study in order to reduce the inter-subject variability, because Gaab et al. [42] observed gender effects in a pitch memory task and Luders et al. [14] showed slight anatomical differences between male and female AP musicians. Although studies frequently restrict their enrollment by gender and age in order to examine a homogeneous group of research participants, we are aware that these kinds of restrictions limit generalizability. Future studies would have to determine whether or not our findings are true for the general population.

*AP testing *– AP was confirmed using an established test [13,48,49] in which participants had to name 52 sine wave tones. Each sine wave tone had an overall duration of 500 ms with an attack and decay rate of 50 ms. The AP test consisted of 13 tones (F#4 to F#5). Each tone was presented four times, resulting in 52 sine wave tones. Participants were instructed to answer as fast and as accurately as possible. In accordance with previous studies [1,48-51], we regarded answers within one semitone of the presented pitch as a correct answer. Since several studies showed that there are different subgroups of AP possessors based on their AP ability (e.g., Miyazaki: precise and imprecise AP group [1]; Itoh et al.: High-, Mid-, and Low AP group [39]), and that these behavioral differences were reflected in electrophysiological responses [39], we decided to use only AP musicians who would belong to either the precise AP group of Miyazaki [1] or the High-AP group of Itoh et al. [39]. Therefore, only AP musicians that scored more than 90% accurate on the AP test were included in the imaging study. The non-AP musicians did not have AP according to their self reports and their performance in the AP test. We only included instrumental musicians in our study. The AP group consisted of 4 keyboard players, 2 string players, and 4 woodwind players, while the non-AP group consisted of 6 keyboard players, 2 string players, and 2 woodwind players. Age of commencement and years of musical training was assessed for every musician.

### Experimental paradigm

**Figure 1:**
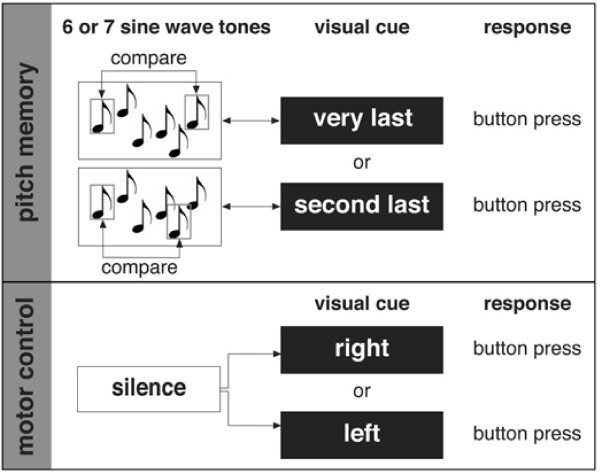
**Schematic description of the experimental task**. Only those tones surrounded by a square correspond to the frequency of the tones of the Western musical scale.

**Figure 2:**
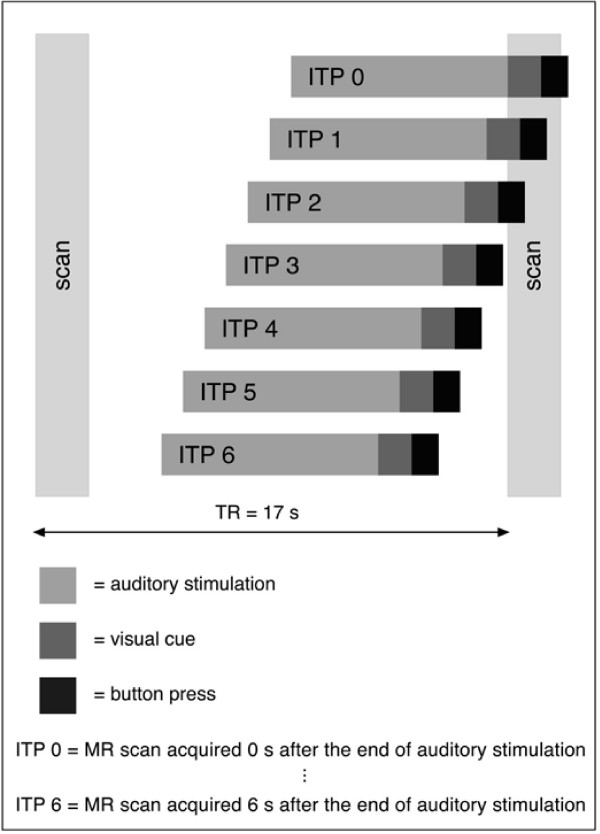
**Jittered sparse temporal sampling technique**. A stack of axial images was acquired over 1.75s every 17s at 6 different imaging time points (ITP) in relation to the task performed. This allowed us to examine the time course of activation and to group ITPs into a perceptual/early encoding phase and a working memory (WM)/multimodal encoding phase.

AP and non-AP musicians were presented with 6- or 7-tone sequences via headphones. All tones were sine wave tones and were generated using the software program Cool Edit Pro (Syntrillium Software). Each tone was 300 ms long with an attack and decay rate of 50 ms and a pause of 300 ms separated each tone from the next, thus resulting in a total duration of 4.6 s for each sequence. Participants were asked to make a decision whether or not the last or second to last tone (as indicated by a visual cue) was "same" or "different" from the first tone. Participants indicated their answers by pressing a button (Fig. 1). Target and probe tones corresponded to the frequencies of tones from the Western musical scale (based on A = 440 Hz) and ranged in frequency from 330 (E4) to 622 (D#5) Hz. The intervening tones, which served as distractor tones, were microtones and did not correspond to fundamental frequencies of the tones from the Western Musical scale (modeled after Deutsch [44]). If the probe was different from the target tone the difference was 2 semitones. The frequency range from the lowest to the highest tone in all sequences was not more than 108 Hz. The use of 6-or 7-tone sequences reduced the possibility that participants could ignore the distractor tones and made the pitch memory task more demanding. The stimulus to imaging time points (ITPs) were kept constant for the 6-and 7-tone sequences (see Fig. 2) by inserting a short silence period prior to the first tone of the 6-tone sequence.

The pitch memory task was contrasted with a motor control condition in which no auditory stimulation took place and participants only pressed the right or left button as indicated by a visual cue (for more details see [37]). All participants were familiarized with the pitch memory task for approximately 10 min prior to the actual MR session using samples of the stimulation material. The behavioral performance during the fMRI session was calculated as a percentage of correct responses/all responses.

### fMRI image acquisition and analysis

Functional magnetic resonance imaging (fMRI) was performed on a Siemens Vision 1.5 Tesla whole-body MRI scanner (Siemens, Erlangen, Germany). Using a gradient-echo EPI-sequence (effective repetition time (TR): 17 s; echo time (TE): 50 ms; matrix size: 64 × 64) a total of 24 axial slices (4 × 4 × 6 mm voxel size) – parallel to the bi-commissural plane – were acquired over 2.75 s each 17 s. In addition, a high-resolution T1-weighted scan (1 mm^3 ^voxel size) was acquired for coregistration with the functional images. We used a variation of a sparse temporal sampling technique [52] with clustered volume acquisition to circumvent interference between the scanner noise and activity of auditory brain regions. The stimulus-to-imaging delay time was varied between 0 to 6 seconds in a jitter-like fashion: Seven different onsets of the auditory sequence relative to the scan were generated, i.e. for ITP 0 the scan started immediately after the end of the auditory sequence, whereas for ITP 6 the scan started 6 s after the end of the auditory sequence (Fig. 2). In using this paradigm we were able to explore the time course of regional fMRI signal changes in response to the perceptual and cognitive demands [37,45] of this pitch memory task (Fig. 2). We applied a box-car function to the fMRI time series and contrasted the pitch memory task with the motor control task. Two subsequent analyses were performed. In the first analysis, we averaged data across all imaging time points (MR acquisitions obtained 0–6 s after the end of the auditory stimulation) and calculated a contrast pitch memory > motor control for each participant and compared the two groups. In the second analysis, we divided the imaging time points (ITP) into two clusters (0–3 s and 4–6 s after the end of the auditory stimulation) making the assumption that the early imaging time points (0–3 s after the end of the auditory stimulation) are more reflective of perceptual and early encoding processes, while the later imaging time points (4–6 s after the end of the auditory stimulation) are more reflective of a WM or other multimodal encoding processes (for more details on this division see also [43]). FMRI data were analyzed using the SPM99 software package http://www.fil.ion.ucl.ac.uk/spm. The MNI coordinates were transformed into the Talairach space [53]. For more details on the analysis of sparse temporal sampling fMRI data see Gaab et al. [37,42]. In order to reveal the entire pattern of differentially activated brain regions (Table 1) and in order to allow comparisons with previous publications, activations were described both at an uncorrected level (*p *< 0.001) and at a corrected level (FDR, p < 0.05). In the discussion we refer to the brain regions that survived only an uncorrected threshold also as regions showing a non-significant trend. To reduce the likelihood of false-positive activations, only clusters containing at least 10 suprathreshold voxels were considered as significant activations [54].

**Table 1:** Activated brain areas during the pitch memory task in AP+ and AP- and the functional activation difference between both groups.

*area*	*left hemisphere*					*right hemisphere*				
	*BA*	*x*	*y*	*z*	*t-value*	*BA*	*x*	*y*	*z*	*t-value*
***AP musicians, ITP 0–3 (cluster level p < 0.05, voxel extent = 10, FDR corr.)***										

Pre-supplementary motor area	**6**	0	8	53	6.6					

inferior parietal lobule	**40**	-50	-38	50	6.32	**40**	53	-34	50	6.91

superior temporal gyrus	**42**	-63	-23	7	14.03	**42**	65	-19	8	13.21

cerebellum		-24	-71	-17	5.19		30 8	-69 -31	-17 -3	7.24 6.23

***AP musicians, ITP 4–6 (cluster level p < 0.05, voxel extent = 10, FDR corr.)***										

inferior frontal gyrus	**44**	-51	15	21	6.52	**45**	51	20	21	5.43

inferior parietal lobule						**40**	48	-48	54	7.79

anterior superior temporal gyrus	**38**	-51	17	-11	7.92					

Cerebellum		-38	-65	-17	6.65		32	-71	-18	7.67

***non-AP musicians, ITP 0–3 (cluster level p < 0.05, voxel extent = 10, FDR corr.)***										

pre-supplementary motor area						**6**	0	14	45	6.21

inferior frontal gyrus						**44**	53	17	25	6.11

inferior parietal lobule						**40**	46	-40	57	8.71

superior temporal gyrus	**42**	-65	-25	12	13.77	**22**	63	-6	-1	12.85

thalamus		0	-11	8	6.71					

Cerebellum		-32	-63	-20	6.93					

***non-AP musicians, ITP 4–6 (cluster level p < 0.05, voxel extent = 10, FDR corr.)***										

inferior parietal lobule						**40**	46	-44	56	7.43

Cerebellum		-36	-63	-19	8.96					

***AP musicians > non – AP musicians, ITP 0–3 (p < 0.001, voxel extent = 10, uncorr.)***										

ventrolateral premotor cortex						**6**	55	7	31	4.78

						**6**	53	2	39	3.69

Precuneus						**7**	2	-76	42	3.43

intraparietal sulcus	**7/40**	-42	-54	51	3.66					

Anterior superior temporal gyrus						**22**	61	-7	11	3.19

						**22**	65	-15	4	4.45

superior temporal sulcus	**21/22**	-61	-18	-4	5.34	**21/22**	67	-35	4	3.67

	**21/22**	-65	-33	7	4.54					

	**21/22**	-63	-23	5	3.56					

brainstem							10	-31	-3	3.55

***AP musicians > non – AP musicians, ITP 4–6 (p < 0.001, voxel extent = 10, uncorr.)***										

IFG	**44**	-50	13	21	3.54	**47**	28	37	-2	3.26

IPCS	**6/44**	-34	5	29	3.41					

precuneus						**7**	4	-54	47	3.54

superior parietal lobule	**7**	-18	-66	49	4.15	**7**	16	-69	51	4

	**7**	-18	-56	43	3.54					

inferior parietal lobule	**40**	-40	-58	51	4.13	**40**	38	-64	47	3.68

	**40**	-46	-33	35	3.67					

***non-AP musicians > AP musicians, ITP 0–3 (p < 0.001, voxel extent = 10, uncorr.)***										

SMA	**6**	0	-15	49	3.43					

postcentral gyrus						**2**	59	-19	40	4.16

postcentral sulcus						**7**	22	-45	69	3.13

Superior parietal lobule	**7**	-14	-51	65	3.59	**7**	30	-45	67	3.8

intraparietal sulcus						**7/40**	40	-42	63	4.57

Inferior parietal lobule	**40**	-61	-39	39	3.21	**40**	36	-33	39	3.3

supramarginal gyrus	**40**	-63	-35	31	3.98					

Superior temporal gyrus	**22**	-59	-24	18	3.58					

inferior temporal gyrus	**20**	-42	-9	-18	3.52					

fusiform gyrus						**37**	44	-45	-13	3.57

thalamus		0	-8	4	4.22					

***non-AP musicians > AP musicians, ITP 4–6 (p < 0.001, voxel extent = 10, uncorr)***										

cingulate gyrus	**32**	-2	10	44	3.39					

postcentral gyrus						**2**	59	-19	40	4.1

superior parietal lobule						**7**	36	-43	65	4.09

intraparietal sulcus						**7/40**	42	-40	61	3.75

superior temporal gyrus	**42**	-63	-23	14	3.78					

	**34**	-24	1	-15	3.32					

anterior superior temporal gyrus	**38**	-38	18	-24	3.48					

Furthermore, a region of interest (ROI) analysis was conducted in order to investigate between-group differences. The cluster of voxels that showed a significant difference (after FDR correction for multiple comparisons, *p *< 0.05) for the early (ITP 0–3) and later processing stage (ITP 4–6) between groups were used to define two ROIs. One ROI was determined by the voxels that showed the strongest activation in the left STS during ITP 0–3 in AP musicians (left STS-ROI, Talairach coordinates: -61 -18 -4, cluster size = 25 voxels, black arrows in Fig. 4) and the second ROI was defined by the strongest activation in the right SPL/IPS region in the non-AP group during ITP 4–6 (right SPL/IPS-ROI, 40 -42 63, cluster size = 17 voxels, black arrows in Fig. 4). Mean regional t-values of these ROIs for every participant (AP and non-AP) and for each imaging time point (ITP 0 through ITP 6) were obtained to visualize the between-group differences over time in more detail (see Fig. 5 and 6).

## Results

### Behavioral Results

The AP group did not differ significantly from the non-AP group in the age of commencement of musical training (AP: 7.8 years (SD 2.86); non-AP: 7.0 years (SD 2.27); *t*(18) = 0.693, *p *= .497) or in the duration of instrumental music training (AP: 15.1 years (SD 3.81); non-AP: 18.8 years (SD 7.41); *t*(18) = 1.38, *p *= 1.83). Although the AP group showed a 96.2% of correct responses (SEM 1.00) in the AP test and the non-AP group's performance was not above chance, performance in the fMRI pitch memory task was not significantly different between groups (AP = 84.5% (SEM 6.0); non-AP = 76.3% (SEM 2.5); *t*(18) = 1.337, *p *= .198). One of the confirmed AP musicians showed only an accuracy of 38% in the fMRI task despite the fact that this individual had an accuracy of 90% in the AP test which employed some of the same sine wave tones. Because this participant performed as accurate as an AP musician in the AP test, it was decided to continue to include him in the fMRI group analysis. Furthermore, this participant did not report any problems before, during or after the fMRI experiment, we assumed that he mixed up the assignment of the "same" and "different" buttons. Within the AP group no significant correlation (two-tailed Pearson Correlation) was observed between the performance in the AP test and the number of correct answers in the experimental pitch memory fMRI task (*r *= .006; *p *= 0.986).

### Functional Imaging Results

**Figure 3:**
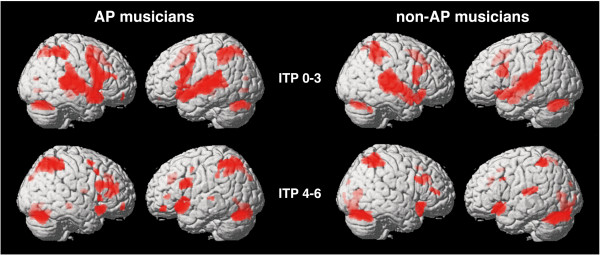
**Activation patterns for AP and non-AP musicians**. Contrasts are separated into early (ITP 0–3) and late (ITP 4–6) imaging time points (*p *< 0.001, uncorrected, voxel extent = 10) superimposed onto the surface reconstruction of a single, normalized brain.

**Figure 4:**
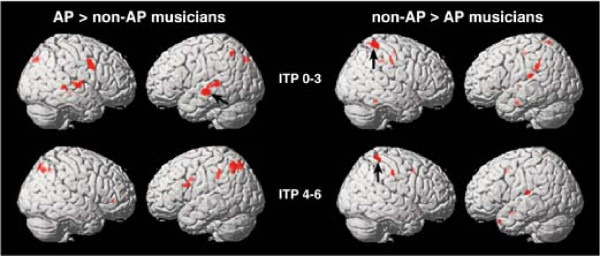
**Between-group contrasts**. The AP versus non-AP as well as non-AP versus AP contrasts are separated into early (ITP 0–3) and late (ITP 4–6) imaging time points (*p *< 0.001, uncorrected, voxel extent = 10) superimposed onto the surface reconstruction of a single, normalized brain. Black arrows indicate the cluster of voxels that survived corrections for multiple comparisons (*p *< 0.05, FDR corrected).

Two statistical thresholds were used to analyze brain activation within groups and between groups. A less conservative threshold (*p *< 0.001, voxel extent = 10, uncorrected for multiple comparisons) was used to show the entire pattern of activated brain regions during the performance of the pitch memory task within each group (Fig. 3) and to show between-group differences (Table 1 and Fig. 4) that would allow comparisons with previous publications. In a second step we applied a more conservative threshold to show only those voxels that survived correction for multiple tests (*p *< 0.05, FDR corrected) (Table 1). Although, our discussion will mainly focus on the significant between group differences (*p *< 0.05, FDR corrected), we will address some of the non-significant trends as well (*p *< 0.001, voxel extent = 10, uncorrected for multiple comparisons). Figure 3 and 4 show the overall activation patterns of both groups and the between-group differences separated into the early perceptual phase (ITP 0–3) and later working memory/multimodal encoding phase (ITP 4–6). For the overall activation pattern, only clusters (cluster level) which showed significant activation (*p *< 0.05, FDR corrected) are reported (Table 1).

During the early imaging time points (ITP 0–3) AP musicians showed bilateral activation of the superior temporal gyrus (STG; BA 42) including Heschl's gyrus (HG), the planum temporale (PT) and extending into the STS, the pre-supplementary motor area (pre-SMA), the inferior parietal lobule (IPL; BA 40) bilaterally, and the superior lateral cerebellum bilaterally. During the later imaging time points (ITP 4–6) AP musicians activated the anterior part of the left STG (BA 38), the inferior frontal gyrus bilaterally (IFG; left BA 44; right BA 45), the right IPL (BA 40), and the cerebellum bilaterally.

The non-AP musicians showed a similar pattern: bilateral activation of the STG (left BA BA 42; right BA 22) extending into the STS and including HG and PT, the pre-SMA, the right IFG (BA 44), the right IPL (BA 40), the left superior lateral cerebellum, as well as the thalamus during the early imaging time points (ITP 0–3). During the later imaging time points (ITP 4–6) significant activations were seen in the right IPL (BA 40) and in the left cerebellum (Table 1).

Figure 4 shows the between-group differences (*p *< 0.001, voxel extent = 10, uncorrected for multiple comparisons) which revealed more STS (ITP 0–3) as well as posterior IPL and posterior IFG (ITP 4–6) activation in the AP>non-AP contrast, while there was more anterior IPL (for ITP 0–3) and SPL (ITP 0–3 and 4–6)) activation in the non-AP>AP contrast. After applying a stricter statistical threshold (*p *< 0.05, voxel extent = 10, FDR corrected), only a region in the middle part of the left STS (Talairach coordinates: -61 -18 -4, cluster = 25 voxels, black arrows in Fig. 4) survived for the AP>non-AP contrast during the early imaging time points (ITP 0–3). No between-group activation differences were observed for the later time points (ITP 4–6). The non-AP musicians showed significantly more activation (*p *< 0.05, voxel extent = 10, FDR corrected) of the right SPL/IPS than the AP musicians (Talairach coordinates: 40 -42 63, cluster = 17 voxels, black arrows in Fig. 4) for all imaging time points (ITP 0–6).

**Figure 5:**
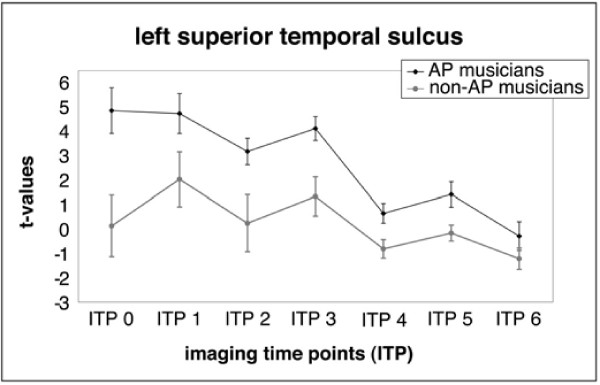
**Time course of mean (SD) regional t-scores for the ROI in the left STS (error bars represent the between subject variability)**.

**Figure 6:**
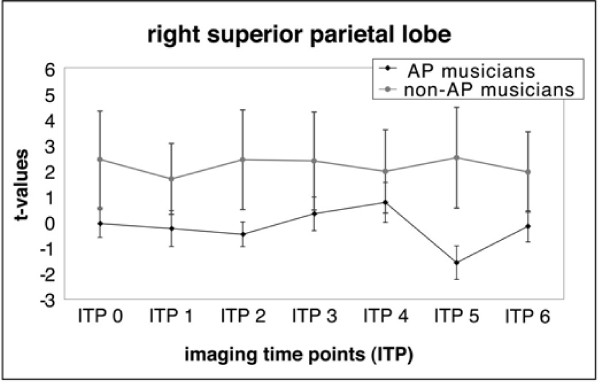
**Time course of mean (SD) regional t-scores for the ROI in the right SPL/IPS (error bars represent the between subject variability)**.

A region of interest (ROI) analysis was done in order to visualize the time course of these between-group differences (see Figs. 5 and 6 for the mean regional *t*-values for both ROIs for each imaging time point). The AP group had higher mean regional *t*-values in the left STS-ROI compared to the non-AP group, especially during the initial imaging time points (ITP 0–3, Fig. 5). The non-AP group had higher mean regional *t*-values than the AP group in the right SPL/IPS-ROI during all imaging time points (ITP 0–6, Fig. 6).

## Discussion

Both groups of musicians had a similar age of commencement and duration (in years) of their musical training, a similar age, gender distribution and played similar instruments, and a similar performance in the fMRI pitch memory experiment, but differed in their AP ability. Thus, common areas of activation in this pitch memory experiment might reflect brain regions that are important for pitch memory performance in general, and between-group differences in the activation pattern will inform us of the neural correlates that are related to the characteristic in which those two groups differed, namely, their absolute pitch ability. The common areas of activation across both groups in this pitch memory task during the early imaging time points included the STG, the IPL, the pre-supplementary motor region, and the superior lateral cerebellum. The common pattern during the later imaging time points included the IPL, and cerebellum. This pattern of activation was also seen in other publications conducting pitch memory experiments [37,43]. One of the main differences between the early and late clusters of imaging time points was the strong temporal lobe activation in the early imaging time points (ITP 0–3) and the weak or absent temporal lobe activation with continued strong activation of extratemporal regions in the frontal and parietal cortex during the later imaging time points (ITP 4–6). This supports our rationale to cluster and divide the imaging time points as we have done, since the early imaging time points (ITP 0–3) might reflect the perceptual and early auditory encoding phase while the later ITPs (ITP 4–6) might reflect higher cognitive processes such as WM [24] and multimodal encoding [55,56].

Differences between the groups (see Table 1 and Fig. 4) were particularly prominent in the left STS (*p *< 0.05, FDR corrected) with trends in the STG on both sides, trends in the posterior IFG and the posterior part of the IPL for the AP>non-AP contrast. The non-AP group differed from the AP group by showing significantly more activation of the right SPL/IPS (*p *< 0.05, FDR corrected) and trends were seen in the anterior part of the left and right IPL.

While the activation pattern of the non-AP musicians reflects the regions of the brain that are active during the early perceptual and tonal memory phase, the activation pattern of the AP musicians might not only reflect the same components, but also regions that are active in encoding tonal information in a unique way (i.e., categorizing tonal information as belonging to pitch chroma classes) and regions that could potentially become active if AP musicians use a verbal label rehearsal strategy to solve the pitch memory task.

In a series of publications, we have shown that the anterior IPL is involved in short-term storage of pitch information in non-musicians as well as in non-AP musicians [35,43,57]. This region has also been found to be involved in short-term storage of verbal auditory information [24,29] and might potentially represent a shared resource for storage of verbal and non-verbal material with possibly some hemispheric differences (depending on whether or not the stored information is verbal and non-verbal, although those hemispheric differences were not seen in our experiment). It is interesting that the activation of the IPL did not differ significantly between the AP and non-AP musicians, although there were some interesting non-significant trends. While non-AP musicians were using more the anterior part of the IPL, AP musicians were showing a trend for more activation of a posterior part of the IPL. We hypothesize that the non-AP musicians might utilize the anterior IPL for short-term storage of tonal information (memorizing the physical characteristics of the tonal information), while the AP musicians might use more the posterior IPL, which could potentially be related to storing tonal information in a different way than non-AP possessors or to store the verbal labels of target tones that have been recognized as belonging to certain pitch categories. Several studies have shown that AP musicians label tones that belong to pitch categories automatically, although this labeling is a learned association and it does not constitute the main difference between AP and non-AP [4,7]. Perceiving tones as belonging to pitch categories, e.g., the early encoding of pitch information, is the main difference between AP and non-AP possessors.

In that context it is interesting that the strongest difference in the AP>non-AP contrast was seen in the left STS region during the perceptual and early auditory encoding phase [43]. Although only the left STS in AP-musicians survived correction for multiple comparisons (*p *< 0.05, voxel extent = 10, FDR correction), the right STS was also more strongly activated compared to non-AP musicians, but no voxels survived the correction for multiple comparisons. Several studies have associated the left (and to a lesser degree the right) STS with the identification or categorization of a variety of sounds (bilateral STS [41,58] and left STS [59,60]), which we postulate is the underlying structure for pitch identification/categorization by AP possessors. Liebenthal et al. [59] found more activation in the middle portion of the left STS (BA 21/22) comparing phonemic with non-phonemic sounds. Similarly important, Mottonen et al. [60], investigating speech perception with 'sine wave speech', found a stronger activation of the left posterior STS when 'sine wave speech' was perceived as speech after a training period as compared to the pre-training period when participants perceived the auditory stimuli as non-speech. The differential activation of this region comparing AP with non-AP musicians is consistent with the hypothesis that AP represents a form of categorical perception [10-12]. The labeling of these pitch chroma categories has been proposed to be the second step in the AP phenotype [7]. The strong left-hemisphere bias in the activation pattern is supported by other studies examining neural correlates of AP [4,15,39].

Results of electrophysiological studies support our neuroimaging findings; even if the timecourse of regional activity changes differs between different neurophysiological and blood-flow dependent brain mapping tools, the temporal sequence of regional activity changes might still be of relevance here. Itoh et al. [39] reported a difference in event-related potentials between musicians with relative pitch (RP) and AP in a tone listening task. They observed an early left posterior temporal negativity in AP compared to non-AP musicians. Hirata and colleagues [38] also found support for an electrophysiological difference between AP and non-AP participants in a passive tone listening task. This electrophysiological characteristic was mapped to the posterior superior temporal plane which has been found to be significantly leftward asymmetric in AP compared to non-AP musicians [3,4,13,14]. Ohnishi et al. [15] found significantly more activation of the posterior superior temporal gyrus region in a passive music listening tasks and related the activity in this region to performance in an AP test. Similarly Wilson et al. [61] found an activation in the left posterior STG/STS region which the authors interpreted as representing a pitch template region in AP possessors. This region might be comparable to the region in the STS that we describe in this manuscript to be more strongly activated in AP compared to non-AP musicians during the perceptual and early encoding phase and which might facilitate the categorization of tonal information in AP possessors.

How is the left PT related to the left STS region? First, there is a wealth of data supporting a reciprocal functional connection between these regions [62-66]. It is possible that the PT serves as an early auditory processing region that influences the processing of auditory information in higher order auditory or multimodal sensory areas in a bottom-up process. Thus, the PT could serve as a hub region that directs the further processing of auditory information [67]. The PT as an auditory association region might process elementary properties of sounds such as pitch height and spectral information [68], while the STS might be involved in the categorization and recognition of sounds based on their elementary properties [68,69]. The pronounced left-sided anatomical asymmetry of the PT in AP musicians [3,4,13,14] might create a functional bias and favour the close interaction between left PT and left STS which could be critical in perceiving tonal information as belonging to pitch chroma categories.

The strongest difference in the non-AP>AP contrast was that non-AP musicians showed significantly more activation of the right SPL/IPS compared to the AP group (*p *< 0.05, voxel extent = 10, FDR corrected). There are two possible explanations for this between-group difference. The first explanation is related to the potential use of a region that is involved in tonal WM. The SPL/IPS has been found to be activated during WM tasks using tonal [35-37] and verbal stimuli [25,27,70], although the strong right hemisphere difference in our data might suggest that the right SPL/IPS could be related more to a tonal WM network. Activity in the SPL/IPS has also been shown to increase as a result of training on a WM task [71]. In addition, Itoh et al. [39] reported that non-AP musicians, while performing a pitch-naming task (reporting vocally the pitches of stimuli as either doh, ray or me), showed three additional ERP components, which were not observed in AP musicians. The authors suggest that the parietal components seen in non-AP participants might reflect relative pitch strategies, possibly through the use of a WM strategy. As described above, several studies showed a smaller or absent P300 during tonal WM tasks in AP [17-20]. Even though the localisation of the P300 proved very difficult [72], and more than one area might serve as the underlying correlate of this component [72], one of the generators of the P300 has been localized in the SPL/IPS region [72,73]. Thus, the increased SPL/IPS activation in the non-AP group might suggest that this region plays a role in the tonal WM network.

However, there is also an alternative interpretation. The SPL/IPS region has been implicated in multimodal sensory integration or encoding [74]. Multimodal sensory encoding and/or integration combined with motor planning, preparation and output is of great importance for an instrumental musician and is practiced throughout their professional career [55,56]. A multimodal encoding strategy such as the utilization of a visual-spatial mapping scheme (i.e., imagining notes on a staff or using a spatial coding for their relative pitch height) for pitch information might be more utilized by the non-AP than the AP musicians which could account for the between-group difference in activation in this region [55,56].

We observed some interesting trends (*p *< 0.001, voxel extent = 10, uncorrected for multiple comparisons) for between-group differences associated with frontal activations that we would like to discuss here, although we want to be careful not to over-interpret these non-significant between-group differences. Most striking was that in the AP>non-AP contrast, we found predominantly stronger posterior IFG activations in the left hemisphere for the later imaging time points (ITP 4–6). The posterior IFG has been implicated in many functions such as sequential auditory tasks and/or predictions of serial auditory events or their violations, but also in pitch and rhythm discrimination tasks and auditory-motor integration functions as they relate to auditory mirror neurons in humans [36,37,75-80]. We are speculating that this observed IFG activation could be unique to the AP phenotype. One of the most obvious explanations is that the left posterior IFG might be related to the rehearsal of verbal labels [24-26,70] and might make this region more part of a verbal WM network as it relates to AP.

Figure 4 suggest that there is slightly more activation of the posterior middle frontal gyrus in the non-AP group compared to the AP group, which could point to an additional region in the tonal memory component of a WM network.

Thus, the unique pattern of activation in the non-AP group in comparison to the AP group might reveal the anatomo-functional network of a tonal memory system as hypothesized by Deutsch [31] which, as we discussed, might show some differences to a verbal memory network, but might also show some shared resources in regions that could store verbal and non-verbal material (e.g., IPL). Further research will be necessary to confirm this interpretation.

The activation of the superior lateral cerebellum did not show any significant between-group difference in the pitch memory task and must therefore be involved in a common process in AP and non-AP participants. We have discussed the role of the cerebellum in previous studies [37] and it is possible that the auditory discrimination component of the pitch memory task, whether or not that was done verbally or physical characteristics of tonal information were used, might explain the cerebellar activation [81,82].

## Conclusion

In summary, the strongest between-group differences in the AP>non-AP contrast were seen in the left STS region possibly reflecting a different perceptual and early encoding process related to the categorization of tonal information into pitch chroma classes. The non-AP>AP contrast showed increased activation in the right SPL/IPS region possibly reflecting a different cognitive strategy in non-AP possessors that might indicate the use of a tonal WM and/or multimodal encoding strategy in order to excel in this pitch memory task.

## Authors' contributions

KS performed the experiment and analysed the data, NG designed the experimental paradigm. KS, NG and GS co-wrote the manuscript. GS supervised the experiment and data analysis. All authors read and approved the final manuscript.
